# Application of Assisted Design of Antibody and Protein Therapeutics (ADAPT) improves efficacy of a *Clostridium difficile* toxin A single-domain antibody

**DOI:** 10.1038/s41598-018-20599-4

**Published:** 2018-02-02

**Authors:** Traian Sulea, Greg Hussack, Shannon Ryan, Jamshid Tanha, Enrico O. Purisima

**Affiliations:** 1 Human Health Therapeutics Research Centre, National Research Council Canada, 6100 Royalmount Avenue, Montreal, Quebec H4P 2R2 Canada; 2 Institute of Parasitology, McGill University, 21111 Lakeshore Road, Sainte-Anne-de-Bellevue, Quebec, H9X 3V9 Canada; 3 Human Health Therapeutics Research Centre, National Research Council Canada, 100 Sussex Drive, Ottawa, Ontario K1A 0R6 Canada; 4 Department of Biochemistry, Microbiology and Immunology, University of Ottawa, 451 Smyth Road, Ottawa, Ontario K1H 8M5 Canada; 5 Department of Biochemistry, McGill University, 3655 Promenade Sir William Osler, Montreal, Quebec H3G 1Y6 Canada

## Abstract

Assisted Design of Antibody and Protein Therapeutics (ADAPT) is an affinity maturation platform interleaving predictions and testing that was previously validated on monoclonal antibodies (mAbs). This study expands the applicability of ADAPT to single-domain antibodies (sdAbs), a promising class of recombinant antibody-based biologics. As a test case, we used the camelid sdAb A26.8, a V_H_H that binds *Clostridium difficile* toxin A (TcdA) relatively weakly but displays a reasonable level of TcdA neutralization. ADAPT-guided A26.8 affinity maturation resulted in an improvement of one order of magnitude by point mutations only, reaching an equilibrium dissociation constant (*K*_D_) of 2 nM, with the best binding mutants having similar or improved stabilities relative to the parent sdAb. This affinity improvement generated a 6-fold enhancement of efficacy at the cellular level; the A26.8 double-mutant T56R,T103R neutralizes TcdA cytotoxicity with an IC_50_ of 12 nM. The designed mutants with increased affinities are predicted to establish novel electrostatic interactions with the antigen. Almost full additivity of mutation effects is observed, except for positively charged residues introduced at adjacent positions. Furthermore, analysis of false-positive predictions points to general directions for improving the ADAPT platform. ADAPT guided the efficacy enhancement of an anti-toxin sdAb, an alternative therapeutic modality for *C. difficile*.

## Introduction

Biotherapeutics including monoclonal antibodies (mAbs) and antibody fragments have become a significant segment of industrial and academic research due to their proven efficacy, safety, and manufacturability profiles^1–3^. Following initial discovery via animal immunization, display-based selection, recombinant library screening, or rational engineering, these molecules typically require subsequent multifaceted optimizations^4^. Structure-based computational approaches can be useful for all major aspects of this optimization phase^5^. Indeed, computational tools have been employed to help improve the binding affinity and specificity^6–9^, immunogenicity^10,11^, and developability^12–14^ profiles of several biologics. Despite some obvious successes, computational methods still face tremendous challenges in predicting these complex phenomena, and the accurate scoring of binding affinity remains a difficult problem^15–19^.

Owing to the importance of affinity maturation to biologics optimization, we have recently devised ADAPT (Assisted Design of Antibody and Protein Therapeutics), a platform that interleaves structure-based computational predictions with experimental testing in order to optimize the binding affinity of a biologic for its target^20^. The strengths of the ADAPT-based affinity maturation rest on: (i) improved consensus-based affinity scoring^21^, and (ii) identifying and eliminating false-positive predictions by experimental testing of top-scored mutants. Importantly, the ADAPT affinity maturation platform is designed to preserve the folding stability of the parent biologic molecule. A current requirement for ADAPT is the availability of 3-D structural data for the protein-protein interface subjected to affinity optimization. Until now, ADAPT affinity maturation has led to 30–100-fold improvements for the binding affinities of several Fab fragments of mAbs that originally bound their antigens with 50 nM–50 pM affinities^20^. However, ADAPT has not been previously utilized for affinity maturation of single-domain antibodies (sdAbs), which are typically characterized by a smaller paratope due to a reduced complementarity-determining region (CDR) relative to conventional mAbs^22,23^. On one hand, smaller antibody-antigen interfaces could lead to weaker binding affinities for sdAbs relative to mAbs, highlighting a need for sdAb affinity maturation (although sdAbs with picomolar antigen-binding affinities have been reported). On the other hand, it is unclear whether the relatively small sdAb paratope can still afford sufficient improvements in binding affinities via the ADAPT platform.

In this study, we examined the applicability of the ADAPT platform to sdAb affinity maturation while continuing our efforts towards improved therapies for *Clostridium difficile* infections (CDI). Treatment of CDI with antibiotics can result in recurrent forms of this highly common and costly hospital-acquired disease^24–26^. With the emergence of antibiotic-resistant and hypervirulent *C. difficile* strains, CDI-associated health-care costs and morbidity rates have prompted alternative treatment modalities including vaccines, fecal transplantation, probiotics and antibody-based immunotherapy^27^. With regard to antibody-based immunotherapy, a number of mAbs targeting and neutralizing *C. difficile* virulence-factor toxins A and B (TcdA and TcdB) have been discovered, with the anti-TcdB mAb bezlotoxumab recently passing a Phase III clinical trial and obtaining FDA-approval^28,29^. SdAbs are also attractive immunotherapeutics, as they present several advantages over mAbs, primarily in terms of ability to access cryptic and concave epitopes, increased stabilities, high modularity and smaller size, thus potentially leading to excellent efficacy, pharmacokinetics and developability profiles^22,23,27,30–34^.

The epitopes of several camelid sdAbs against TcdA and TcdB have been recently delineated by X-ray crystallography^35^. One of the most interesting of these anti-toxin sdAbs is the A26.8 V_H_H, shown to recognize a unique epitope on the extreme C-terminus of the receptor-binding domain (RBD) of TcdA. Although A26.8 binds to TcdA with a weaker affinity than A20.1 (another V_H_H that binds TcdA at multiple epitopes non-overlapping with that of A26.8), A26.8 is a more potent neutralizer of TcdA than A20.1^36^. These properties led to the selection of A26.8 as our candidate for ADAPT optimization with the goals of improving its TcdA binding affinity and enhancing its toxin neutralization capacity.

## Methods

### *In silico* affinity maturation

The atomic coordinates of the A26.8 V_H_H bound to the C-terminal portion of TcdA were taken from the structure of the A26.8H6-TcdA-A2 complex crystallized at pH 6.5 (PDB ID: 4NC0)^35^, and were used as a starting point for virtual affinity maturation (see also associated protein sequences and residue numbering in Supplementary Fig. S1). Two versions of the complex were prepared, differing by exclusion (preparation 1) or inclusion (preparation 2) of the C-terminal G262 of TcdA and N-terminal Q1 of the V_H_H, which are not visible in the crystal structure but may affect the calculated interactions in the complex. All TcdA amino-acid residues preceding A123, which are distant from the V_H_H, and the His-tag residue H125 at the C-terminus of the V_H_H, were deleted from the crystal structure. Hydrogen atoms were added to the resulting complex and adjusted for maximizing H-bonding interactions. Structural refinement of the complex was then carried out by energy-minimization using the AMBER force-field^37,38^, with a distance-dependent dielectric and an infinite distance cutoff for non-bonded interactions. Non-hydrogen atoms were restrained at their crystallographic positions with harmonic force constants of 40 and 10 kcal/(mol.A^2^) for the backbone and side-chain atoms, respectively.

The ADAPT platform was then used for affinity maturation^20^. In the first round of affinity optimization, single-point scanning mutagenesis simulations were carried out at several positions within the CDRs of V_H_H A26.8. We used three protocols, SIE-SCWRL^39–41^, FoldX^42,43^, and Rosetta^44,45^, for building the structures and evaluating the energies of single-point mutations to 17 other possible natural amino acids (Cys and Pro were excluded) at these positions of the parental sequence. A consensus approach over specific versions of these three protocols was applied for building and scoring the V_H_H mutants. Scoring of binding affinity was mainly based on the average Z-score and also on the average rank score over the scores calculated with the three component energy functions, SIE^40,41^, FoldX-FOLDEF^42^, and Rosetta-Interface^44^. Further technical and implementation details of this consensus approach and its component methods can be found in Sulea *et al*.^21^. Prior to binding affinity predictions, the FoldX-FOLDEF energy function^42^ was used to estimate the effect of substitutions on the internal stability of the V_H_H structure. Thus, mutations predicted to be destabilizing by introducing folding free energy changes larger than 2.71 kcal/mol (i.e., 100-fold increase of unfolding equilibrium constant) relative to the parental molecule were discarded from further evaluation. In the second round of optimization, double- and triple-point V_H_H mutants were generated from combinations of the lead single-point mutations selected after experimental validation, and scored using the same computational protocol as for the single-point mutants.

### Protein expression and purification

The DNA sequences of A26.8 V_H_H mutants were synthesized commercially by Thermo-Fisher/GENEART (Regensburg, Germany), subcloned into the pSJF2H expression vector^46^, with C-terminal Myc/His6 tags, and were subsequently expressed in *E. coli*. Briefly, 50 ng of plasmid DNA was added to 5 µL of Mix and Go TG1 *E. coli* competent cells (Zymo Research, Irvine, CA) according to the manufacturer’s instructions before plating onto 2 × YT/ampicillin agar plates and incubation overnight at 32 °C. For expression, 5 mL 2 × YT cultures containing 100 µg/mL ampicillin, 1% (w/v) glucose were inoculated with single plasmid-bearing *E. coli* colonies and grown overnight at 37 °C with shaking at 220 rpm. The next day, 1 mL of overnight culture was inoculated into 250 mL 2 × YT/ampicillin, 0.1% (w/v) glucose, in 500 mL baffled Ultra Yield flasks (Thomson Instruments Inc., Oceanside, CA) with air top seals and grown until an OD_600_ ~ 0.5–0.8. Cultures were then induced with 200 mM IPTG and grown overnight at 37 °C with shaking at 220 rpm. Periplasmic-targeted V_H_Hs were extracted by osmotic shock and supernatants filtered through 0.22 µM filters (Millipore, Etobicoke, ON, Canada). V_H_Hs were purified by immobilized metal-ion affinity chromatography (IMAC) using Ni Sepharose^TM^ excel affinity resin (GE Healthcare, Mississauga, ON, Canada) in binding buffer containing PBS pH 7.4, 400 mM NaCl, and eluted in buffer containing PBS pH 7.4, 400 mM NaCl, 250 mM imidazole.

### Binding affinity measurements

Before surface plasmon resonance (SPR) binding experiments, IMAC-purified V_H_Hs were subjected to de-salting and further purification by size-exclusion chromatography (SEC). Approximately 500 µg of each V_H_H was injected over a Superdex 75 Increase 10/300 GL SEC column (GE Healthcare) in Biacore running buffer HBS-EP (10 mM HEPES, pH 7.4, 150 mM NaCl, 3 mM EDTA, 0.005% (v/v) P20; GE Healthcare) under the control of an ÄKTA FPLC at 0.8 mL/min. Monomeric V_H_H fractions were collected and analyzed for binding to TcdA using a Biacore 3000 SPR instrument (GE Healthcare). Approximately 4,500 resonance units (RUs) of TcdA (List Biological Laboratories, Campbell, CA) were immobilized on a CM5 sensor chip (GE Healthcare) using the conditions previously described^36^. HBS-EP running buffer was used for all binding studies and regeneration of TcdA surfaces. Next, various dilution ranges of V_H_Hs (as low as 10–0.5 nM to as high as 5 µM–50 nM) were injected over immobilized TcdA at a flow rate of 40 µL/min with 120 s contact time and 600 s dissociation time. Reference-subtracted sensorgrams were analyzed with BIAevaluation software (GE Healthcare) and fit to a 1:1 binding model. In cases where rapid *k*_on_ and *k*_off_ rate constants were observed, equilibrium dissociation constants (*K*_D_s) were determined by steady-state analysis. All mutant A26.8 V_H_Hs were run in duplicates and the parent A26.8 V_H_H in quintuplicate. The discrepancies in the *k*_on_, *k*_off_ and *K*_D_ values reported for wild-type A26.8 V_H_H in this study *vs*. our previous study^36^ (*k*_on_ = 1.8 × 10^6^ *vs*. 1.4 × 10^6^ M^−1^ s^−1^, *k*_off_ = 3.4 × 10^−2^ *vs*. 1.6 × 10^−2^ s^−1^ and *K*_D_ = 19 *vs*. 12 nM) are due to the modification to protocols and reagents used in this work.

### Thermal stability measurements

Differential scanning fluorimetry (DSF) was used to determine the melting temperatures (*T*_m_) of the parental and mutant A26.8 variants. DSF was carried out in a Rotor-Gene 6000 real-time PCR instrument (Corbett Life Science, Mortlake, NSW, Australia). Samples were diluted in HyClone™ Dulbecco’s phosphate-buffered saline (D-PBS; GE Healthcare) at a final concentration, after mixing, of 0.33 mg/mL. A total volume of 30 μL in 0.2 mL thin wall PCR tubes (Axygen, Oneonta, NY) was used. SYPRO® Orange (Life Technologies, Burlington, ON, Canada) was diluted 1,000-fold from the 5,000x concentrated stock to the working dye solution in D-PBS and 15 μL were added to 15 μL of sample just prior to the experiment. Thermal denaturation was carried out by increasing the temperature from 30 °C to 94 °C at a rate of 0.06 °C/s. Fluorescence intensity, with excitation at 470 nm and emission at 610 nm, was collected at 1 °C intervals and analyzed with the Rotor Gene 6000 series software v1.7 (Corbett Life Science). The *T*_m_ values were determined from the peak of the first derivative transformation of the raw data. Each sample was measured in quadruplicate.

### Toxin A inhibition assays

Vero cells (CCL-81; ATCC, Manassas, VA) were maintained in complete media (MEM + antibiotic/antimycotic + 10% FBS) in T-75 flasks at 37 °C, 5% CO_2_. For TcdA inhibition assays, sterile 96-well tissue culture plates (Thermo-Fisher, Ottawa, ON, Canada) were seeded with ~2 × 10^4^ cells/well in a total volume of 200 µL of complete media. Plates were incubated for 24 h at 37 °C, 5% CO_2_. Media was then carefully removed from each well and replaced with 200 µL of fresh media, 20 µL of antibody (serially diluted in sterile PBS, giving final in-well concentrations ranging from 1 µM to 1.95 nM) and 10 µL of TcdA (List Biological Laboratories; final in-well concentration of 10 ng/mL corresponding to 32.5 pM) for all wells. This concentration of TcdA was 10-fold lower than that used on in our earlier work^36^ and reflects the differences in TcdA potency among suppliers and the increased sensitivity of Vero cells to TcdA relative to human fibroblast cells (data not shown). Control wells received 20 µL of PBS instead of antibody, or 10 µL of PBS instead of TcdA. Plates were then incubated at 37 °C and 5% CO_2_ for 72 h. Next, the media was removed and replaced with 100 µL of pre-warmed MEM containing 10% WST-1 cell proliferation reagent (Roche, Laval, QC, Canada). Plates were incubated at 37 °C and 5% CO_2_ for 1 h before reading the absorbance at 450 nm using a Multiskan^TM^ FC photometer (Thermo-Fisher). Data were analyzed for % inhibition of TcdA (relative to untreated control wells) and graphed using GraphPad Prism software. The reported values are mean TcdA inhibition derived from four independent assays.

## Results

### First ADAPT cycle–single-point mutants

**Figure 1:**
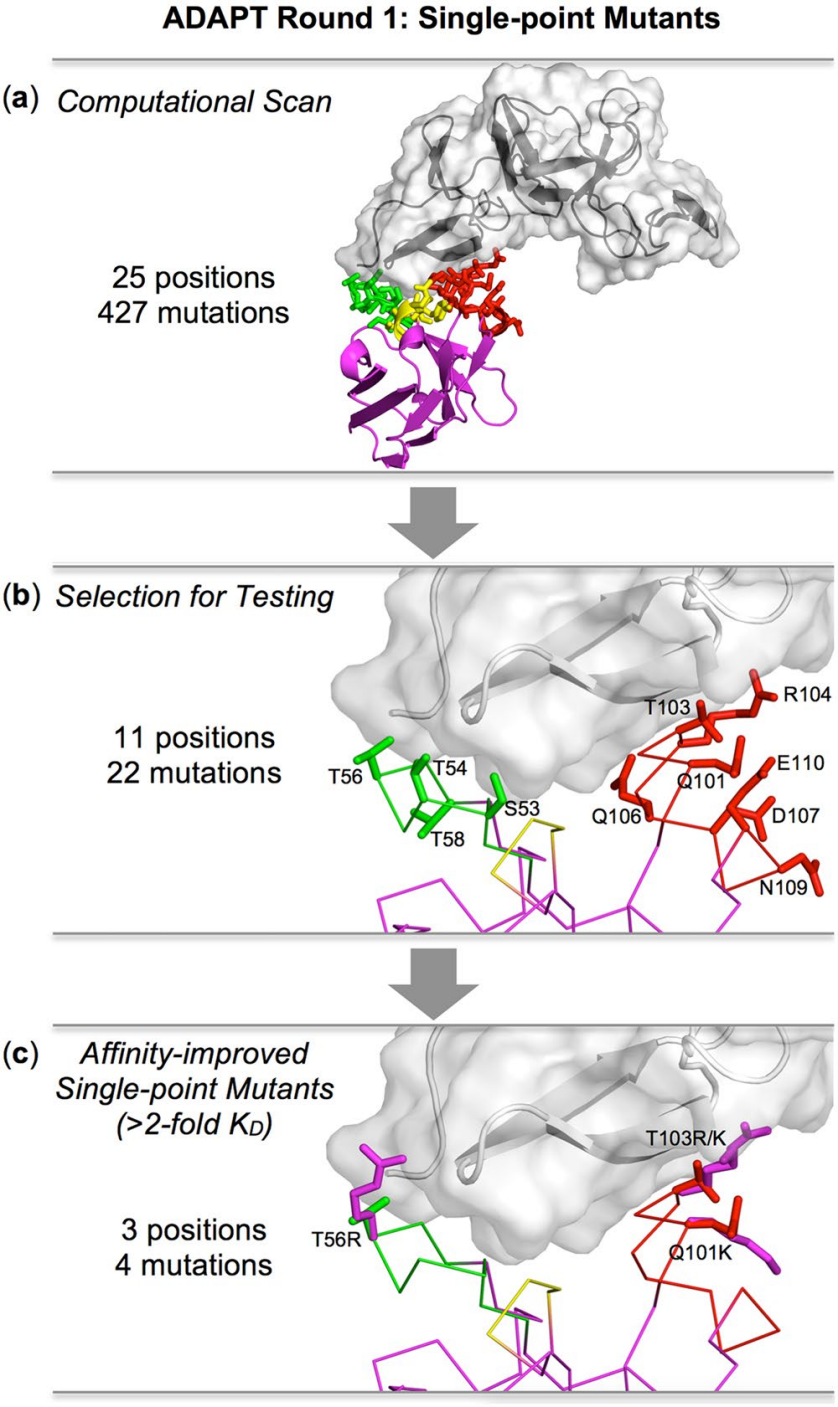
Round 1 of ADAPT leading to validated affinity-improved single-point mutants of a *C. difficile* TcdA-binding V_H_H. (**a**) Proximity of virtually scanned CDR residues to the TcdA epitope. (**b**) 3-D locations of positions where mutations were selected for experimental testing. (**c**) Single-point mutations found to lead to antigen binding affinity improvements of over 2-fold relative to parental V_H_H. The antigen is shown as black/gray ribbon in a translucent molecular surface. The V_H_H is rendered as a magenta ribbon/Cα-trace, with CDR1 in yellow, CDR2 in green, and CDR3 in red, and their side chains shown as stick models. Models of mutated side-chains are shown in (**c**) as magenta sticks.

In the first round of ADAPT, 427 single-point mutations to all natural amino acids except Cys and Pro were computationally evaluated in the CDRs of V_H_H A26.8. The scanned region covered 25 positions (S30-P33 from CDR1, V50-Y59 from CDR2, and S100-E110 from CDR3) that when substituted have the potential to alter the antigen-binding affinity. The proximity of the targeted residues to the TcdA fragment can be seen in Fig. 1a.

The selection of the most likely single-point mutants with improved antigen-binding affinities was primarily guided by the top 50 consensus Z-scores based on predictions from the three computational methods within ADAPT. These cover 13 positions and show only minor differences between the results obtained with the two structural preparations described in the Methods section (Table 1 versus Supplementary Table S1). Additional information was gleaned from the top 50 single-point mutations sorted by the average rank of the three component methods of ADAPT. Although these largely overlap with the mutations ranked by the consensus Z-score, there are several differences with the notable addition of 2 new positions (T58 and N109) (Supplementary Tables S2 and S3). Visual examination of the molecular interactions predicted with the three sampling protocols of ADAPT (SCWRL, Rosetta, FoldX) was used to detect sub-optimal complementarity at the antibody-interface (e.g., burial of polar groups in non-polar environments), steric overcrowding and distortions in covalent geometry (e.g., deviations from planarity of aromatic rings). This qualitative process narrowed down the selection to a set of 22 single-point mutations at 11 positions for further evaluation. These point mutations can be classified in two groups according to their location relative to the antigen and the interaction type (Table 2). The “Contact” set includes 15 substitutions at 6 positions contributing to the main contact surface interacting with the antigen, whereas the 7 mutations forming the “Peripheral” set substitute 5 positions located more at the periphery of the antibody-antigen interface (Fig. 1b). The mutation T103M was not listed in the top-50 mutations by either the consensus Z-score or average rank, but was selected based on a favorable FoldX binding affinity score and the prediction of good structural complementarity to the antigen surface.

**Table 1:** Top 50 consensus Z-scores for single mutants.

Residue	R	K	Q	N	S	T	H	W	Y	F	M	L	I	V	A	G	E	D
S53	**−0.9**																	
T54	**−1.6**							**−1.4**	**−1.1**	−1.1								
T56	**−1.7**												**−1.2**					
S57				−1.0														
Y59												−1.5						
Q101					−0.9													
T103	**−1.5**	**−1.5**	−1.0					**−1.6**										
R104			−1.0	−1.3				**−5.2**	−3.3	−3.4		−0.9						
Q106								**−1.9**		**−1.2**		**−1.4**	−0.9				−0.9	
D107	**−1.5**	−1.1	−1.3	**−1.1**	−1.1	−1.1	−1.2	−1.4	**−1.5**	−1.5	−1.2	−1.2	−1.2	−1.2	−1.1	−1.1	−1.3	
P108	−1.0	−1.1						−1.0	−1.0	−1.0	−1.0				−0.9			
E110			**−1.0**															

Structure preparation 2 was used, which includes modeled N-terminal residue Q1 of V_H_H and C-terminal residue G262 of TcdA. Single-point mutations experimentally tested are highlighted in bold. Parent antibody Z-score = −0.33.

**Table 2:** Summary of the ADAPT-based selection for single-point mutants of V_H_H A26.8 with predicted increased affinity for toxin A^a^.

Mutation group	CDR2 mutations	CDR3 mutations
Contact	S53R T54R, T54W, T54Y T56R, T56I	T103R, T103K, T103W, T103M^c^ R104W Q106W, Q106F, Q106Y, Q106L
Peripheral	T58K^b^	Q101K^b^ D107R, D107Y, D107N N109R^b^ E110Q

^a^From top-50 consensus Z-scores and visual inspection unless otherwise specified.

^b^From top-50 average rank scores and visual inspection.

^c^Based on the FoldX binding score and visual inspection.

**Figure 2:**
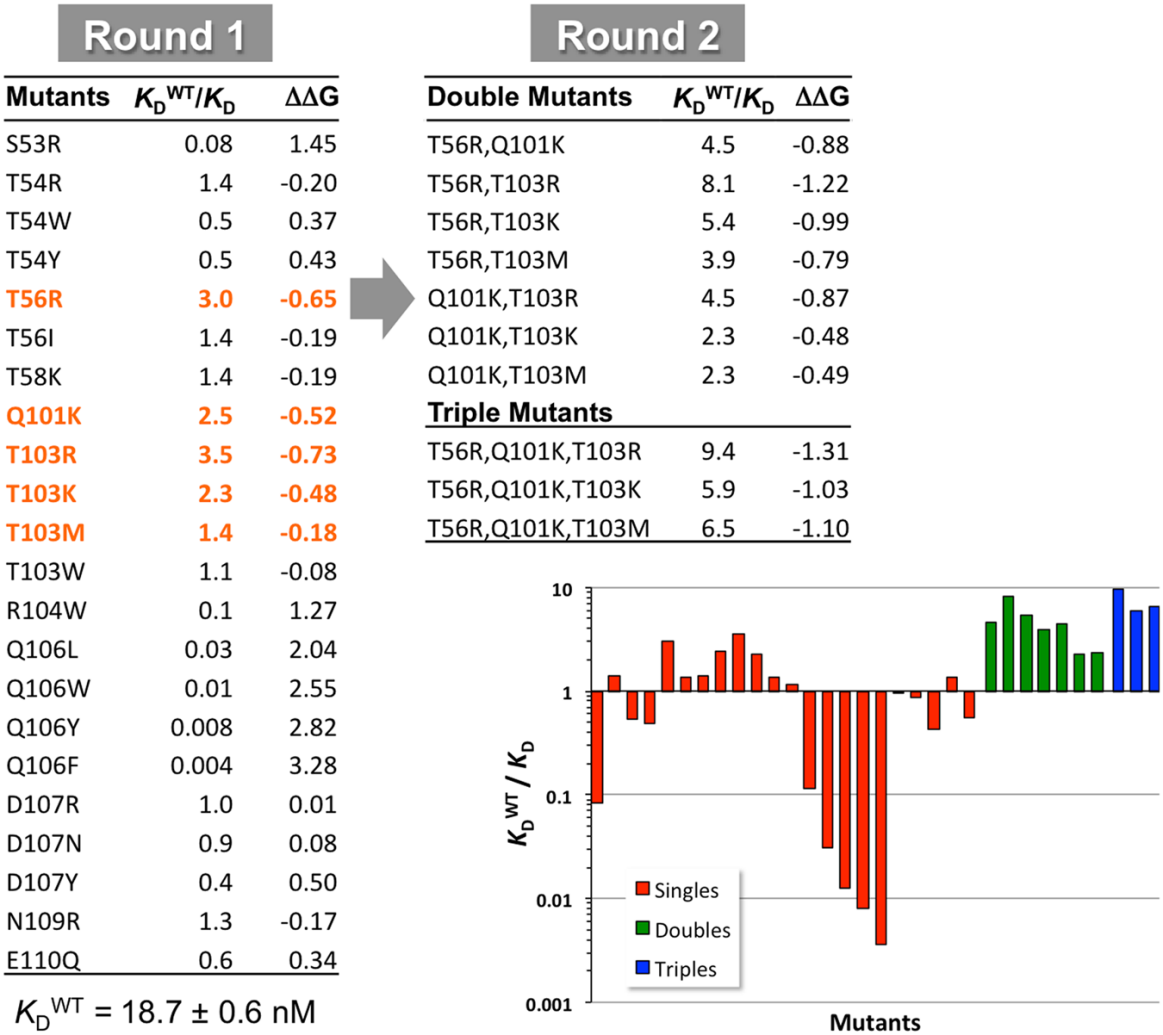
Ratios of wild-type to mutant *K*_D_s (*K*_D_^WT^/*K*_D_) and differences in binding free energies (ΔΔG, kcal/mol) relative to the parent V_H_H during two rounds of mutations. Round 1 mutants that were carried forward to Round 2 are highlighted in orange.

These 22 single-point mutants were then produced and tested comparatively with the parental 26.8 V_H_H. The *K*_D_s and kinetic constants (*k*_on_ and *k*_off_) measured by SPR are listed in Table 3. Improvements in binding affinities relative to the parent A26.8 V_H_H are presented in Fig. 2. Almost half of the single-point mutants demonstrate improved binding affinities, with two mutants (T56R and T103R) showing about a 3-fold improvement, and another two (Q101K and T103K) over 2-fold. The location of these mutations relative to the antigen epitope is illustrated in Fig. 1c. Substitution of T103 with the hydrophobic residues Met and Trp led to smaller improvements than with Arg and Lys. Similarly, a smaller improvement was obtained by substituting T56 with Ile rather than Arg. Mutations T58K and N109R, considered based on their average rank scores, only marginally improved antigen binding. The same marginal improvement was also observed for the T54R mutation; however, T54 substitution with aromatic amino acids decreased affinity. Mutations that were predicted to improve antigen-binding affinity but had the opposite effect included mutations at positions D107 and E110 (up to 2.5-fold decrease in affinity), at S53 and R104 (about 10-fold decrease in affinity) and at Q106 (30–300-fold decrease in affinity).

**Table 3:** Binding and stability analyses of A26.8 V_H_H mutants.

V_H_H	*k*_on_ (10^6^) (M^−1^ s^−1^)^a^	*k*_off_ (10^−2^) (s^−1^)^a^	*K*_D_ (nM)^a^	*T*_m_ (°C)^b^
*Parent A26.8*	1.8 (0.3)	3.4 (0.6)	18.7 (0.6)	78.2 (0.1)
*Round 1 mutants*				
T103R	2.7 (1.0)	1.4 (0.3)	5.3 (0.8)	79.1 (0.1)
T56R	4.8 (2.8)	2.7 (1.0)	6.2 (1.7)	77.2 (0.1)
Q101K	2.7 (0.9)	2.0 (0.2)	7.6 (1.9)	81.4 (0.1)
T103K	2.5 (1.1)	2.0 (0.6)	8.3 (1.2)	78.5 (0.1)
T54R	3.6 (1.8)	4.4 (1.1)	13.2 (3.6)	75.5 (0.0)
T58K	2.5 (0.7)	3.3 (0.3)	13.5 (2.3)	76.9 (0.2)
T56I	1.9 (0.5)	2.6 (0.5)	13.6 (1.0)	76.5 (0.0)
T103M	1.8 (0.5)	2.4 (0.5)	13.7 (1.5)	77.4 (0.1)
N109R	2.0 (0.2)	2.7 (0.01)	13.9 (1.6)	77.6 (0.1)
T103W	2.4 (1.0)	3.6 (0.4)	16.3 (5.5)	77.5 (0.0)
D107R	1.4 (0.3)	2.6 (0.1)	19.0 (3.3)	70.8 (0.5)
D107N	1.9 (0.5)	4.0 (0.6)	21.3 (2.1)	74.9 (0.1)
E110Q	2.1 (0.4)	7.0 (1.6)	33.6 (1.8)	78.1 (0.1)
T54W	1.6 (0.2)	5.6 (0.0)	35.0 (3.7)	74.0 (0.6)
T54Y	1.8 (0.4)	6.9 (0.2)	38.9 (6.8)	76.0 (0.1)
D107Y	1.6 (0.3)	7.1 (0.9)	44.0 (1.3)	73.4 (0.1)
R104W	0.3 (0.0)	5.5 (0.8)	165 (9)	77.3 (0.0)
S53R^c^			224 (170)	74.0 (0.0)
Q106L^c^			612 (53)	80.0 (0.0)
Q106W^c^			1475 (177)	79.7 (0.1)
Q106Y^c^			2315 (473)	79.0 (0.0)
Q106F^c^			5130 (1174)	81.0 (0.0)
*Round 2 mutants*				
T56R, Q101K, T103R	12.3 (4.6)	2.4 (0.8)	2.0 (0.1)	81.6 (0.1)
T56R, T103R	8.3 (0.9)	1.9 (0.2)	2.3 (0.0)	78.5 (0.1)
T56R, Q101K, T103M	10.4 (2.0)	3.0 (0.2)	2.9 (0.3)	80.9 (0.1)
T56R, Q101K, T103K	14.7 (3.6)	4.7 (1.1)	3.2 (0.1)	81.3 (0.1)
T56R, T103K	8.6 (3.3)	2.9 (0.9)	3.4 (0.3)	78.5 (0.1)
T56R, Q101K	12.4 (3.2)	5.0 (0.3)	4.1 (0.8)	81.4 (0.1)
Q101K, T103R	5.5 (0.7)	2.3 (0.2)	4.2 (1.0)	81.9 (0.1)
T56R, T103M	6.4 (1.1)	3.0 (0.3)	4.8 (1.3)	77.0 (0.0)
Q101K, T103M	3.3 (0.1)	2.7 (0.1)	8.0 (0.6)	81.3 (0.0)
Q101K, T103K	5.0 (1.3)	4.0 (0.3)	8.2 (1.7)	81.5 (0.0)

^a^Mean (±standard deviation) from n = 2 experiments, except for the parent V_H_H (n = 5).

^b^Mean (±standard deviation) from n = 4 samples.

^c^*K*_D_s determined by steady-state affinity analysis.

In this first ADAPT round consisting of single-point mutants, there was no clear trend as to whether *k*_off_ or *k*_on_ was more affected by the mutations. For example, in the case of the best two single-point mutants, T103R seems to benefit from an improvement in *k*_off_ whereas T56R from an improvement in *k*_on_ (about 2.5-fold relative to the parent in each case). Importantly, most of the first-round single-mutants had thermal stabilities similar to the parental A26.8 V_H_H with a *T*_m_ of about 78 °C (Table 3). This was expected since the FoldX stability scores were used to filter out mutations predicted to be significantly destabilizing. Mutations Q101K, T103R and those at position 106 had a stabilizing effect relative to the wild-type (Supplementary Fig. S2). The most destabilizing effect was observed for the D107R mutant; however its *T*_m_ still remained above 70 °C.

### Second ADAPT cycle–double and triple mutants

The four lead single-point mutations from round 1, possessing greater than 2-fold improvements in *K*_D_s, were carried forward to round 2 of affinity maturation. These include T56R in CDR2, and Q101K, T103R and T103K in CDR3. Combination of these mutations can lead to a total of 5 double mutants and 2 triple mutants. Because of the proximity of positions 101 and 103, that are both substituted with positively charged amino acids, we also included in this round the charge-neutral mutation T103M, which led to a slight affinity improvement in the first round, hence adding 2 double and 1 triple mutants to the second set. These 10 mutants considered in the second round were first verified computationally within ADAPT, mainly for potential changes in folding stability as a result of adjacent mutations. All mutants were predicted to have either improved or similar stabilities relative to the parental V_H_H. Relative changes in binding affinity were also calculated with the ADAPT protocols, which generally predicted additive improvements relative to the component single mutations. Exceptions included the non-additivity predicted for the double- and triple-mutants that had simultaneous introduction of two basic residues in CDR3 (Q101K and T103R/K); nevertheless these mutations were predicted to improve affinity relative to the parent molecule. In these cases, introduction of Met at position 103 was predicted to restore some additivity and hence supported its consideration for the second round of optimization.

Determination of the melting temperatures for the 10 double and triple mutants indicates that 7 have increased stabilities, 2 have similar stabilities and one has a slightly decreased stability relative to parental A26.8 (Table 3, Supplementary Fig. S2). The increase in thermal stability can be associated with the presence of the Q101K mutation, consistent with the ~3 °C increase in *T*_m_ seen for this single-point mutant.

**Figure 3:**
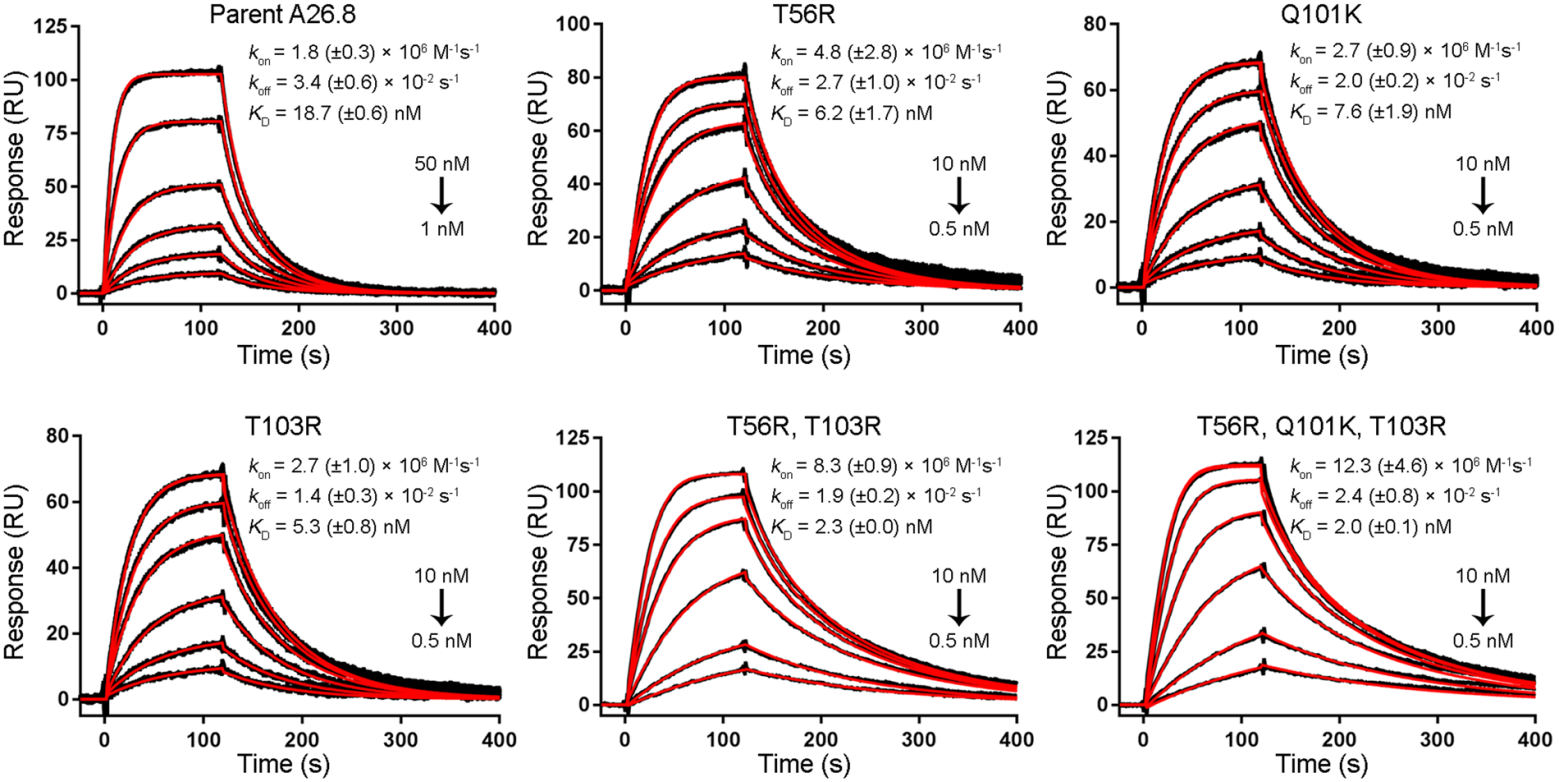
Representative SPR sensorgram binding profiles. Interaction of the parent A26.8 V_H_H, the lead single mutants T56R, Q101K and T103R, the lead double mutant T56R, T103R, and the lead triple mutant T56R, Q101K, T103R with immobilized full-length TcdA. The black lines represent raw data and the red lines are global fits to a 1:1 bimolecular interaction model. See the Methods section for experimental details. Mean (±standard deviation) values are shown along with the V_H_H concentration range used in each experiment.

The antigen binding kinetic and affinity constant data determined by SPR measurements are presented in Table 3 and Fig. 2. All 10 multiple mutants show improved binding affinities relative to the parent A26.8 V_H_H antibody, and 8 of them also show affinity improvements relative to the best single-point mutant, T103R. However, it should be noted that the second cycle of ADAPT did not afford further improvements in the dissociation rate constant (*k*_off_) relative to the best single-point mutant, T103R. The best double mutant is T56R,T103R combining the two best single mutations, which occur in CDR2 and CDR3. The 8-fold affinity improvement comes from an over 4-fold increase in *k*_on_ coupled with a small 1.8-fold decrease in *k*_off_, relative to the parent A26.8 V_H_H. The improvement in binding affinity does not come at the cost of stability. The best mutant tested (T56R, Q101K, T103R) is a triple mutant that incorporates the beneficial Q101K mutation into the best double mutant. This leads to an overall 10-fold improvement of *K*_D_, with an ~7-fold effect attributed to the change in *k*_on_, as well as a 3.4 °C increase in *T*_m_ relative to the original V_H_H A26.8. Examples of SPR sensorgrams are shown in Fig. 3 for the best double and triple mutants comparatively to those of the component single mutants and the parent molecule, visually illustrating changes in the kinetic behavior that are favorable to the mutants. Not only are the best multiple mutants composed of the best single mutants, but there is also a generally robust additivity of mutation effects on binding affinity throughout the entire data set (Supplementary Fig. S3).

### *In vitro* toxin neutralization by lead variants

**Figure 4:**
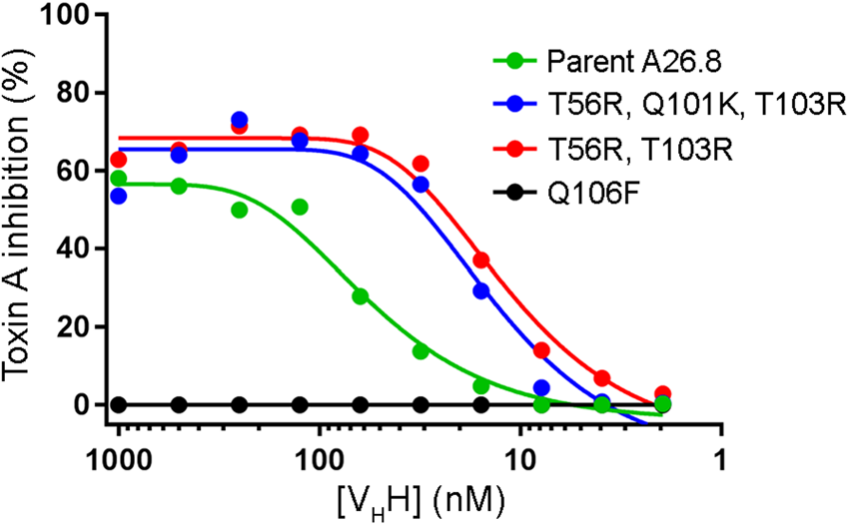
*In vitro* TcdA neutralization assay with affinity-matured V_H_Hs. Vero cells were incubated with TcdA (10 ng/mL = 32.5 pM) and increasing concentrations of V_H_Hs. Cell proliferation was monitored spectrophotometrically relative to untreated controls and cells receiving TcdA only. Plotted are mean data from four independent assays.

The antigen-binding affinity improvements obtained at the end of the second cycle of ADAPT prompted us to test the best binders for TcdA neutralization at the cellular level. TcdA-induced Vero cell cytotoxicity data as a function of V_H_H concentration are shown in Fig. 4. It is clear that the best affinity-matured variants (double mutant T56R, T103R and triple mutant T56R, Q101K, T103R) have an increased protective effect against *C. difficile* TcdA relative to the parent V_H_H. In this assay, both mutants show about the same TcdA IC_50_ of ~12 nM versus ~70 nM for the parent A26.8 V_H_H, a 6-fold increase in neutralization potency. It is also interesting to note that the affinity-matured variants can reach maximal TcdA inhibition levels around 70% (at 60 nM V_H_H), whereas the parent V_H_H can only reach ~60% and that at a much higher concentration (500 nM). As a negative control, we also tested one of the single-point mutants with reduced antigen binding affinity, Q106F, which as expected failed to inhibit TcdA cytotoxicity (Fig. 4). Taken together, these data demonstrate that ADAPT-guided optimization of the V_H_H paratope for improved antigen-binding affinity can translate into a marked enhancement of TcdA neutralization at the cellular level. These data also demonstrate the transferability of the ADAPT affinity maturation platform from mAbs/Fab fragments to sdAbs.

## Discussion

With the refinement of binding affinity prediction algorithms and growth of structural data for protein-protein complexes, the rational structure-based approach is poised to become a tractable choice for affinity maturation of protein-protein interfaces. The field of affinity maturation has been traditionally dominated by established methods like display-based selection and functional screening of large, random or site-directed libraries^47–49^. Extensive use of these methods, particularly for affinity maturation of antibodies, indicates typical affinity improvements in the 10–1000-fold range. The limited number of applications of the structure-driven ADAPT platform to affinity maturation thus far showed a performance in the 10–100-fold range^20^. ADAPT also provides a structural framework for rationalization of affinity improvements at the atomic level. Presently, the main limitation of the structure-driven approach rests with the need for detailed 3-D structural knowledge of the protein-protein interface (preferably from a crystal structure). Improvements in protein-protein docking algorithms with optional utilization of epitope mapping constraints (e.g., from NMR experiments or mutagenesis data) give hope for eliminating this requirement in the future.

An important objective of this work was to explore the applicability of the ADAPT affinity maturation platform to sdAbs, ADAPT having been successfully applied to several mAb-antigen interfaces in earlier campaigns. Initial concerns were raised due to the smaller paratope of sdAbs relative to the two-chain variable domain binding site of conventional mAbs, which reduces the number of antigen contacts and limits the mutational space available for ADAPT-guided affinity maturation. The present ADAPT campaign began with an sdAb that had a relatively weak binding affinity to its antigen (*K*_D_ ≅ 20 nM). It is reasonable to expect that relatively weak antigen-binding affinities are not uncommon for sdAbs, in particular those isolated from synthetic/semi-synthetic and naïve sdAb libraries, although many picomolar sdAbs have been reported from immune libraries. Here, despite the reduced paratope area, ADAPT was able to guide sdAb affinity maturation to an improvement of about one order of magnitude. Thus, one finding of this study is that an sdAb paratope can afford binding affinity improvements by point substitutions only, i.e., no insertion/deletion required, by applying the ADAPT affinity maturation platform. Another finding is that this level of affinity improvement translated into significant activity enhancements at the cellular level. However, the reduced mutational space available within the CDRs of sdAbs and its impact on the extent of ADAPT affinity maturation cannot be underestimated. An illustration comes from the ADAPT affinity maturation of the engineered bH1 Fab that binds VEGF with relatively weak affinity (*K*_D_ ≅ 40 nM) similar to that of the A26.8 V_H_H–TcdA complex^20^. In that system, ADAPT was able to reach over 100-fold improvement of binding affinity, which can at least in part be attributed to the increased mutational space available within the six Fab CDR loops relative to the three found in sdAbs.

**Figure 5:**
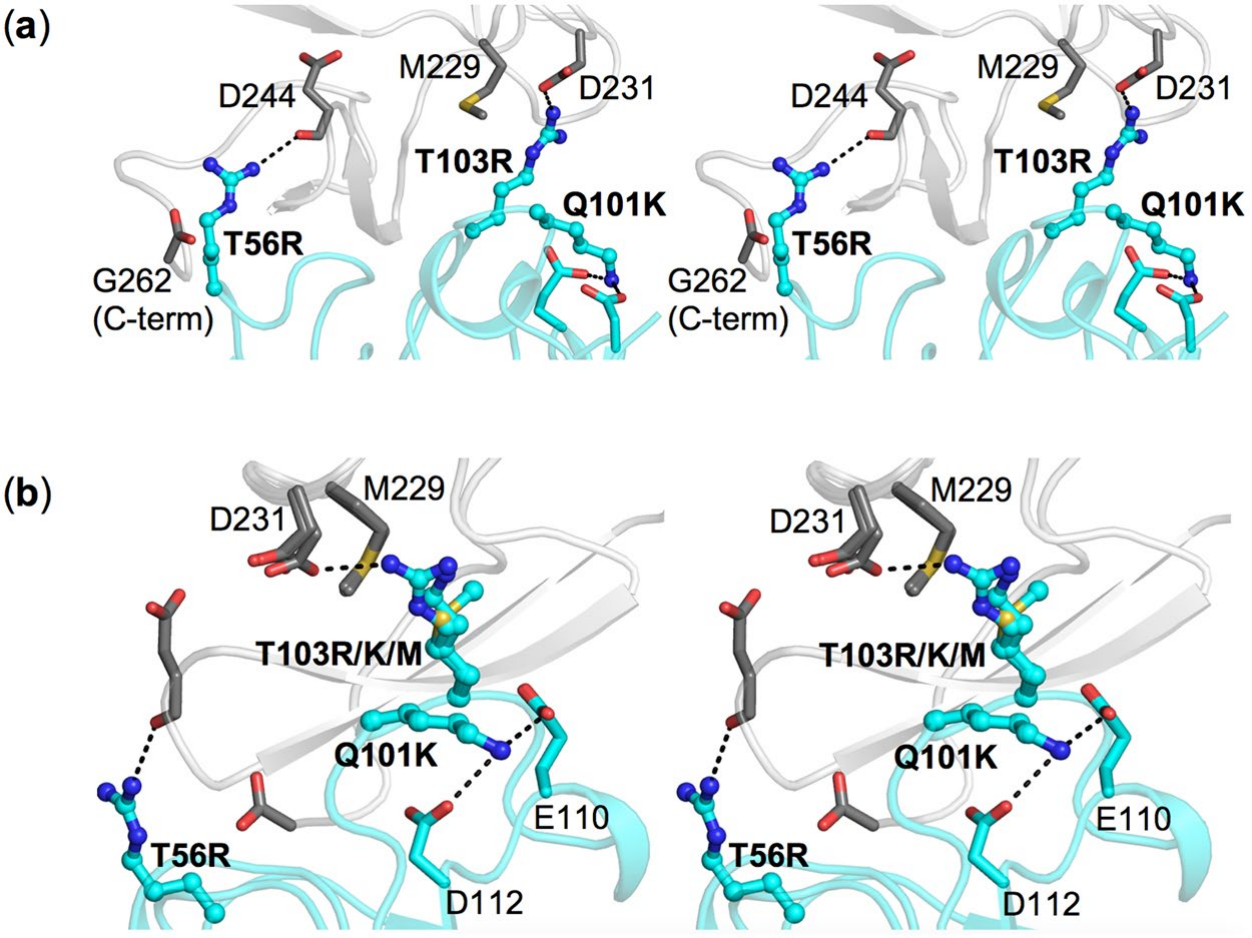
Molecular models of optimized TcdA-V_H_H interactions. (**a**) and (**b**) Represent stereoviews showing from different angles the interactions at mutated positions 56, 101 and 103 of the V_H_H. Panel (b) additionally includes an overlay of three different mutations at position 103. The TcdA is rendered in gray and the V_H_H in cyan. H-bonds are indicated by black dashed lines.

One of the advantages of rational structure-guided affinity maturation is that it helps understand the structural basis for improvement of binding affinity. In Fig. 5a we display the atomic-level interactions predicted for triple mutant T56R,Q101K,T103R with the TcdA fragment. It can be seen that both of the main contributors to the affinity improvement, the arginines at positions 56 and 103, establish novel, relatively short-range electrostatic interactions with negatively charged groups of the antigen (D231, D244, and G262 C-terminus). The lysine at the more peripheral position 101 seems to act by neutralizing some of the negative charges on the sdAb itself (at E110 and D112 of CDR3) hence alleviating some of the long-range electrostatic repulsion to the antigen present in the parental molecule. The new intramolecular H-bond interactions introduced by Lys at position 101 (Fig. 5) are also likely responsible for the observed increase in stability upon the Q101K mutation (Table 3, Supplementary Fig. S2). The aliphatic portion of R103 may also benefit from novel short-range contacts with M229 of the antigen. As shown in the rotated view of Fig. 5b, the hydrophobic Met side chain at position 103 could still contact M229 of the antigen, but lacks the potential of Arg or Lys for electrostatic interactions, thereby leading to a smaller improvement in binding affinity (Fig. 2, Table 3). Between the two positively charged side-chain substitutions at position 103, Arg is favored according to the SPR measurements, in agreement with structural predictions showing that the shorter Lys side chain does not reach close enough to D231 of the antigen in order to establish an H-bonding interaction as in the case of R103 (Fig. 5b).

It is equally important to analyze the major false-positive predictions. They include all four hydrophobic substitutions at position Q106, as well as S53R and R104W (Fig. 2, Table 3). Substitutions of Q106 with Trp, Phe, Tyr or Leu had the most detrimental effects on antigen binding affinity, yet their calculated consensus scores were reasonably high (Table 1, Supplementary Table S1) and were well-accommodated at the binding interface (Supplementary Fig. S4). A closer examination of the interactions established in the parent complex highlights the presence of water-mediated H-bond interactions between the side-chain amide moiety of Q106 and the main-chain NH group of L240 of the antigen (Supplementary Fig. S5). Explicit water-mediated interactions and the cost of displacing structured water molecules may not have been properly captured by the continuum treatment applied here. This points to a possible direction towards improving the current computational protocol in ADAPT. R104W was predicted with a very favorable Z-score (Table 1, Supplementary Table S1), but was not present in the top-50 by the average rank score (Supplementary Tables S2 and S3). Since this mutation was predicted as very favorable by the Rosetta method only, revisiting the ranking protocol relative to the Rosetta-Interface scoring function, in which the electrostatic component is significantly downscaled, may be further explored in the future. Qualitatively, the interactions established by W104 appear feasible, including an H-bond with the main chain carbonyl group of A237 of the antigen (Supplementary Fig. S4). The S53R mutation was predicted to be only marginally improving, based on R53 establishing H-bond interactions with E242 of the antigen differently than the parental S53 (Supplementary Fig. S4); the structural basis for its detrimental effect as measured by SPR remains elusive.

There is no requirement or guarantee for additivity of mutation effects on binding affinity; however, multiple-point mutants benefiting from additivity are those generally leading to the largest improvements in binding affinity. Mutation additivity of affinity improvements also serves to validate the single-mutation data and increases the confidence in the observed trends. In the present sdAb affinity maturation data set, the additivity regression slope and intercept are close to what was previously observed from the ADAPT affinity maturation of several mAbs^20^. We observed that most of the additive mutations are not mutually influencing each other, which may have contributed to the maximal additivity obtained by combining these mutations. Mutually interacting mutations (often at adjacent 3-D positions) might reduce additivity, or even eliminate it totally to the point of decreased affinity relative to the parent, for example by steric collisions between the mutated side-chains. Within the present set of sdAb mutants, those incorporating simultaneous substitutions of the adjacent positions 101 and 103 with positively charged amino acids benefit somewhat less from additivity (blue symbols in Supplementary Fig. S3) than the other multiple mutants. This partial loss of additivity was in fact expected as it was inferred at the earlier stage of computational prediction. In contrast to the strong additivity of affinity improvements, no additive effects of *k*_off_ improvements were observed, and in fact the *k*_off_ improvements obtained for the single mutants relative to the parent were small (less than 2-fold except for T103R). This led to binding affinity improvements for the multiple mutants that were largely dominated by *k*_on_ increases (Table 3). Since these mutants include substitutions to charged residues, improving binding affinities via enhanced association rates is an expected behavior^50^. However, despite a relatively poor *k*_off_ of the parental V_H_H and marginal *k*_off_ improvements afforded by ADAPT, it is noteworthy that the obtained *k*_on_ improvements were able to significantly enhance the inhibition potency at the cellular level.

The results reported here also have implications towards improved biotherapeutics against *C. difficile* infections. As noted earlier, sdAbs represent alternative therapeutic modalities to antibiotics that induce resistance, disease recurrence and incur high costs. They also have largely recognized advantages over mAbs in terms of size, epitope accessibility and stability. In this study, ADAPT-guided affinity maturation helped improve the potency of the A26.8 V_H_H for neutralization of *C. difficile* TcdA, a proven virulence factor of CDI. The observation that binding affinity correlated with neutralization potency lends further support to the notion that the unique A26.8 epitope located at the extreme C-terminus of TcdA-RBD is highly functional and perhaps implicated directly in toxin contacts with the host cell receptor. It should be noted that the lead A26.8 mutant was only capable of a maximum of 70% TcdA inhibition, and at an EC_50_ concentration of 12 nM the V_H_H is present in ~400-fold molar excess of TcdA. Regardless, many other high-affinity sdAbs show no toxin inhibition, and other sdAbs display weaker inhibition potency despite having high valency and stronger binding affinity (e.g., the A20.1 V_H_H)^27,35,36^. Because the A26.8 epitope on TcdA was shown to be non-overlapping with those of several other neutralizing sdAbs, including A20.1, it is expected that combining the improved A26.8 variants reported here, e.g., the T56R,T103R double mutant, with those sdAbs will impart further increases in toxin neutralization potency, as previously shown for the parental A26.8 V_H_H^36^.

## Electronic supplementary material

Supplementary Information
