# Supplementary material for: Application of Assisted Design of Antibody and Protein Therapeutics (ADAPT) improves efficacy of a Clostridium difficile toxin A single-domain antibody
Source: Sci Rep. 2018 Feb 2;8:2260. doi: 10.1038/s41598-018-20599-4 (PMC5797146; doi:10.1038/s41598-018-20599-4)
Supplement: Supplementary file 1 — Supplementary Information [file 41598_2018_20599_MOESM1_ESM.pdf]

Supplementary Information

for

# Application of Assisted Design of Antibody and Protein Therapeutics (ADAPT) improves efficacy of a *Clostridium difficile* toxin A single-domain antibody

Traian Sulea[1], Greg Hussack[2], Shannon Ryan[2], Jamshid Tanha[2,3], Enrico O. Purisima[1,*]

[1]*Human Health Therapeutics, National Research Council Canada, 6100 Royalmount Avenue, Montreal, Quebec, Canada, H4P 2R2*

[2]*Human Health Therapeutics, National Research Council Canada, 100 Sussex Drive, Ottawa, Ontario, Canada, K1A 0R6*

[3]*Department of Biochemistry, Microbiology and Immunology, University of Ottawa, 451 Smyth Road, Ottawa, Ontario, Canada, K1H 8M5*

* Corresponding author: enrico.purisima@nrc-cnrc.gc.ca

**Supplementary Table S1.** Top 50 consensus Z-scores for single mutants.

| Res | R | K | Q | N | S | T | H | W | Y | F | M | L | I | V | A | G | E | D |
|---|---|---|---|---|---|---|---|---|---|---|---|---|---|---|---|---|---|---|
| V50 | | | | | | -0.9 | | | | | | -1.3 | | | | | | |
| S53 | -0.8 | | | | | | | | | | | | | | | | | |
| T54 | -0.8 | | | | | | | | | | | | | | | | | |
| T56 | -1.1 | | | | | | | | | | | | -0.8 | | | | | |
| Y59 | | | | | | | | | | -1.2 | | -1.4 | -0.8 | | | | | |
| Q101 | | | | | -0.8 | -0.8 | | | | | | | | | | | | |
| T103 | -1.2 | -1.0 | | | | | | -1.1 | | | | | | | | | | |
| R104 | | | | -1.0 | | | | -4.3 | -2.5 | -2.6 | | | | | | | | |
| Q106 | | | | | | | | -1.7 | -1.1 | -1.1 | | -1.3 | -0.8 | | | | | |
| D107 | -1.4 | -1.1 | -1.1 | -1.0 | -1.0 | -1.0 | -1.1 | -1.3 | -1.4 | -1.3 | -1.0 | -1.1 | -1.1 | -1.1 | -1.0 | -1.0 | -1.1 | |
| P108 | -1.0 | -1.0 | -0.9 | | | | | -0.9 | -0.9 | -0.9 | -0.9 | -0.9 | | | -0.9 | | | |
| E110 | | | -1.0 | | | | | | | | | | | | | | | |

Structure preparation 1 was used, which does not include the N-terminal residue Q1 of $V_H$H and C-terminal residue G262 of TcdA. Single-point mutations experimentally tested are highlighted in red. Parent antibody Z-score = -0.26.

**Supplementary Table S2.** Average ranks using ADAPT on the structure preparation 2.

| Res | R | K | Q | N | S | T | H | W | Y | F | M | L | I | V | A | G | E | D |
|---|---|---|---|---|---|---|---|---|---|---|---|---|---|---|---|---|---|---|
| Y32 | | 100 | | | | | | | | | | | | | | | | |
| I51 | | | | | | | | | | | | 100 | | | | | | |
| T54 | 77 | | | | | | | | 93 | 89 | | | | | | | | |
| T56 | 30 | | | | | | | | | | | | 59 | | | | | |
| T58 | | 90 | 83 | | | | | 94 | 102 | 89 | | 77 | 89 | 85 | | | 98 | |
| Y59 | | | | | | | | | | 72 | | 95 | | | | | | |
| T103 | 68 | 54 | | | | | | 26 | | | | | | | | | | |
| Q106 | | | | | | | | 93 | | 81 | | 45 | | | | | 88 | |
| D107 | 67 | 92 | 89 | 85 | 91 | 86 | 68 | 63 | 53 | 50 | 91 | 83 | 93 | 84 | 92 | 97 | 69 | |
| P108 | | 93 | | | | | | 59 | 63 | 58 | 85 | | | | 84 | | | |
| N109 | 102 | | | | | | | | | | | | | | | | | |
| E110 | | | 94 | | | | | | | | | | | | | | | |

Single-point mutations experimentally tested are highlighted in red.

**Supplementary Table S3.** Average ranks using ADAPT on the structure preparation 1.

| Res | R | K | Q | N | S | T | H | W | Y | F | M | L | I | V | A | G | E | D |
|---|---|---|---|---|---|---|---|---|---|---|---|---|---|---|---|---|---|---|
| S30 | 99 | 93 | | | | | | | | | | | | | | | | |
| Y32 | | 84 | | | | | | | | | | | | | | | | |
| I51 | | | | | | | | | | | | 91 | | | | | | |
| T54 | 94 | | | | | | | | | | | | | | | | | |
| T56 | 49 | | | | | | | | | | | | 93 | | | | | |
| T58 | | 67 | | | 87 | | | | | | | | | 96 | | | | |
| Y59 | | | | | | | | | | 52 | | 46 | | | | | | |
| Q101 | | 82 | | | | | | | | 90 | | 95 | 78 | | | | | |
| T103 | 80 | 100 | 101 | | | | | 92 | | | | 92 | | | | | | |
| Q106 | | 93 | | | | | | 80 | 54 | 42 | | 39 | 95 | 101 | | | | |
| D107 | 81 | 95 | 97 | 97 | | 100 | 76 | 73 | 55 | 56 | 100 | 91 | | 95 | | | 87 | |
| P108 | | 99 | | | | | | 89 | 92 | 84 | 92 | 92 | | | 84 | | | |
| N109 | 90 | | | | | | | | 87 | | | | | | | | | |

Single-point mutations experimentally tested are highlighted in red.

## A26.8 $V_HH$

*4NC0 structure numbering (this work)*

10 20 30 40 50 60 70 80 90 100 110 120

QVKLEESGGGLVQAGGSLRLSCAASERTFS**RYPVA**WFRQAPGAEREFVA**VISSTGTSTYYADSVKG**RFTISRDNAKVTVYLQMNNLKREDTAVYFCAV**NSQRTRLQDPNEYDY**WGQGTQVTVSS

CDR1 CDR2 CDR3

## TcdA C-terminal fragment

*4NC0 structure numbering (this work)*

130 140 150 160 170 180 190 200 210 220 230 240 250 260

AATGWVTIDGRRYYFEPNTAIGANGYKIIDNKNFYFRNGLPQIGVFKGPNGFEYFAPANTDANNIDGQAIRYQNRFLHLLGNIYYFGNNSKAVTGWQTINGNMYYFMPDTAMAAAGGLFEIDGVIYFFGVDGVKAPGIYG

2580 2590 2600 2610 2620 2630 2640 2650 2660 2670 2680 2690 2700 2710

*Full-length TcdA numbering*

**Supplementary Figure S1.** Amino acid sequences of the protein fragments used for *in silico* affinity maturation studies. The residue numbering adopted throughout this work corresponds to those from crystal structure with PDB code 4NC0. Also provided for TcdA is the residue numbering corresponding to the full-length toxin. The sequence of the $V_HH$ A26.8 used for experimental measurements in this work has a C-terminal Myc-His6 tag (GSEQKLISEEDLNHHHHHH) that was not included for the *in silico* studies. The three CDR loops of the $V_HH$ are highlighted. Residues tested experimentally during the first round of ADAPT are marked by empty circles below the A26.8 VHH sequence, with the three residues that progressed to the second round of affinity maturation indicated by filled circles.

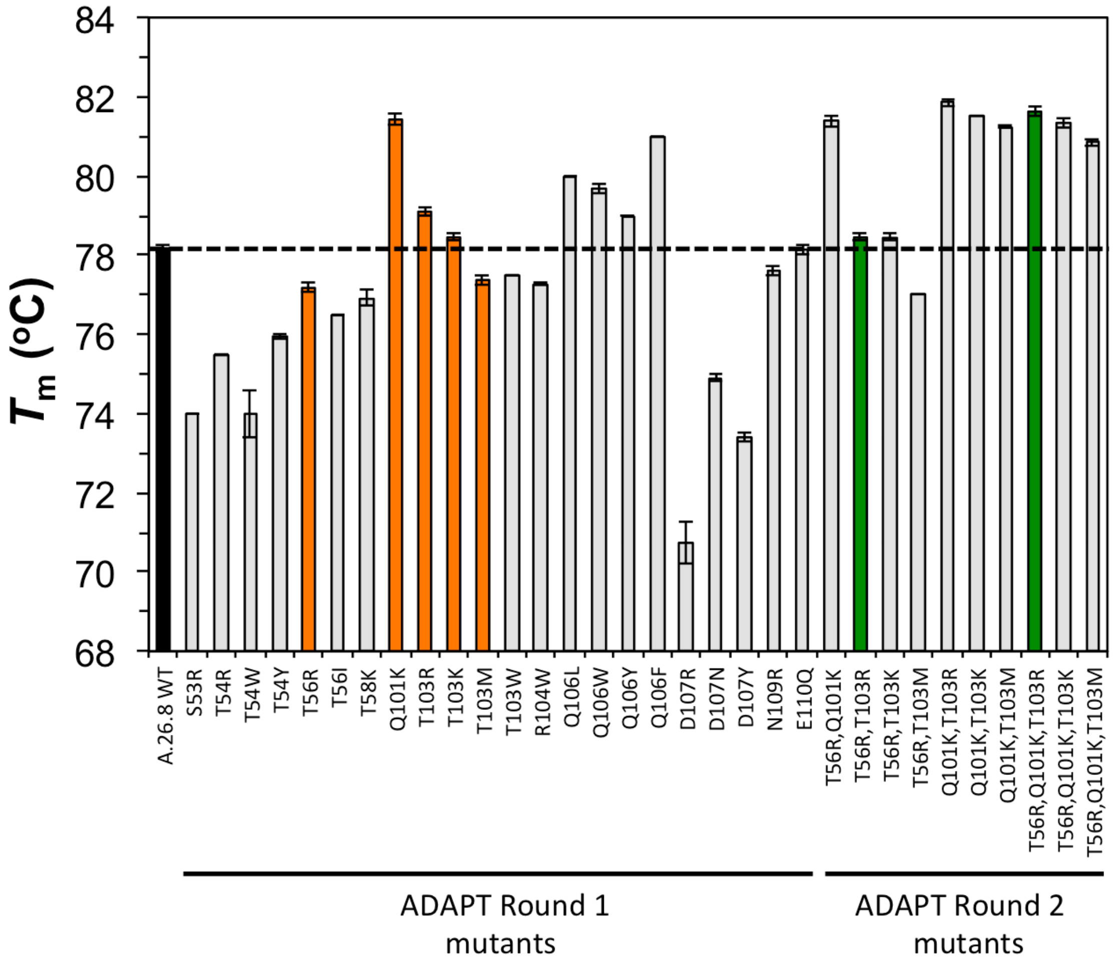


**Supplementary Figure S2.** Overview of thermal stabilities expressed as melting temperatures ($T_m$s) measured by DSF for the A26.8 $V_H$H variants analyzed in this study. Dashed line represents the $T_m$ of the wild-type A26.8 $V_H$H. Single mutants carried forward to the second round of ADAPT are highlighted in orange. Improved-affinity mutants tested in cellular assays for inhibition of TcdA cytotoxicity are highlighted in green. See the Methods section for experimental details.

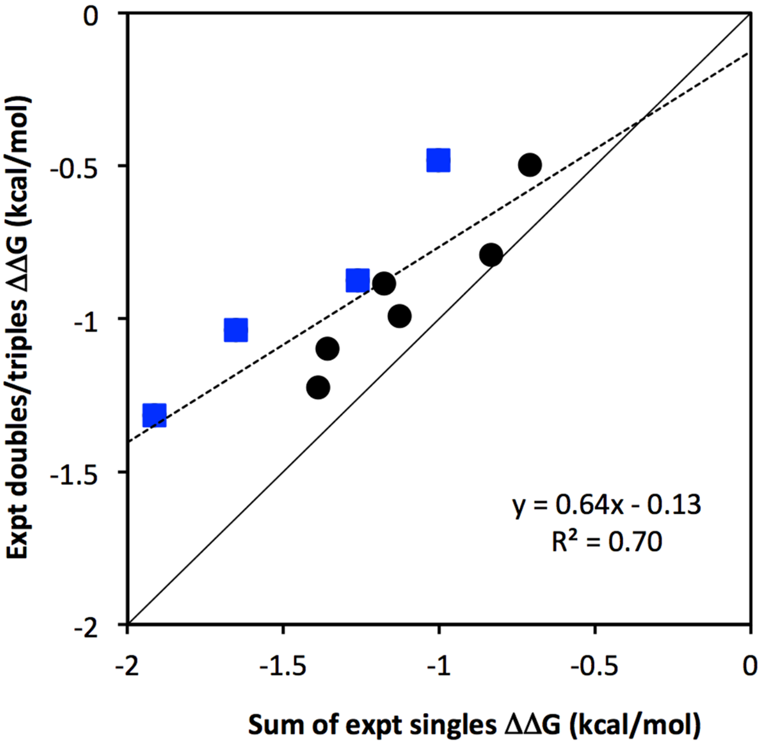


**Supplementary Figure S3.** Additivity of contributions of mutations to binding affinity. Scatter plot of experimentally measured relative binding affinities of double and triple mutants versus the sum of experimentally measured relative binding affinities of the component single mutants. The dashed line is the linear regression line for the entire data set, while the solid diagonal line indicates full additivity. Blue symbols highlight mutants incorporating simultaneous substitutions at adjacent positions 101 and 103 with positively charged amino acids.

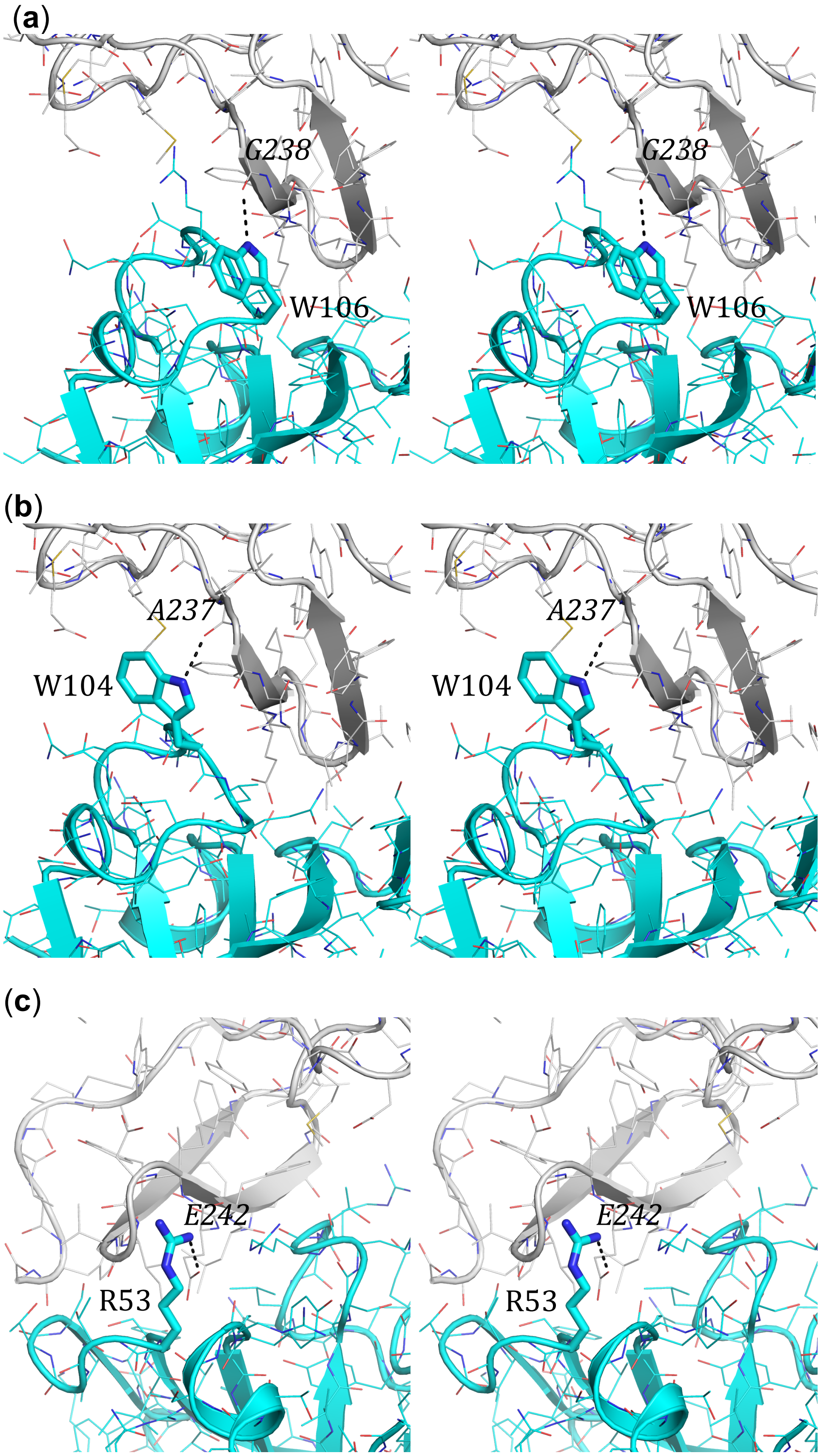


**Supplementary Figure S4.** Stereoviews of interactions around mutated sites for the major false positives. (a) Q106W mutation; (b) R104W mutation; (c) S53R mutation. The TcdA antigen fragment is rendered in gray and the antigen-bound A26.8 $V_HH$ in cyan with the mutated residues shown as thick sticks. H-bonds to TcdA residues (labeled in italics) are indicated by black dashed lines.

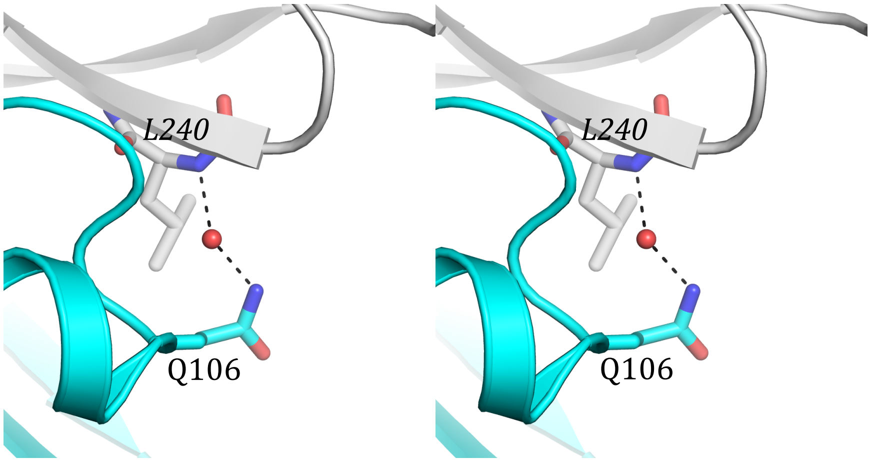


**Supplementary Figure S5.** Stereoview of a water-mediated interaction between the side chain of Q106 from $V_H$H A26.8 and the main chain of L240 from TcdA. Coordinates are taken from the PDB entry 4NC0. The TcdA antigen fragment is rendered in gray and the antigen-bound A26.8 $V_H$H in cyan. The bridging water molecule is shown as a red sphere. Intermolecular H-bonds are indicated by black dashed lines.
